# N-acetyl cysteine prevents arecoline-inhibited C2C12 myoblast differentiation through ERK1/2 phosphorylation

**DOI:** 10.1371/journal.pone.0272231

**Published:** 2022-07-28

**Authors:** Yi-Xuan Li, Chun-Hung Hsiao, Yung-Fu Chang

**Affiliations:** 1 Department of Biomedical Science and Environmental Biology, Kaohsiung Medical University, Kaohsiung, Taiwan; 2 Translational Research Center of Neuromuscular Diseases, Kaohsiung Medical University Hospital, Kaohsiung, Taiwan; 3 Department of Medical Research, Kaohsiung Medical University Hospital, Kaohsiung, Taiwan; University of Tennessee Health Science Center College of Graduate Health Sciences, UNITED STATES

## Abstract

Arecoline is known to induce reactive oxygen species (ROS). Our previous studies showed that arecoline inhibited myogenic differentiation and acetylcholine receptor cluster formation of C2C12 myoblasts. N-acetyl-cysteine (NAC) is a known ROS scavenger. We hypothesize that NAC scavenges the excess ROS caused by arecoline. In this article we examined the effect of NAC on the inhibited myoblast differentiation by arecoline and related mechanisms. We found that NAC less than 2 mM is non-cytotoxic to C2C12 by viability analysis. We further demonstrated that NAC attenuated the decreased number of myotubes and nuclei in each myotube compared to arecoline treatment by H & E staining. We also showed that NAC prevented the decreased expression level of the myogenic markers, myogenin and MYH caused by arecoline, using immunocytochemistry and western blotting. Finally, we found that NAC restored the decreased expression level of p-ERK1/2 by arecoline. In conclusion, our results indicate that NAC attenuates the damage of the arecoline-inhibited C2C12 myoblast differentiation by the activation/phosphorylation of ERK. This is the first report to demonstrate that NAC has beneficial effects on skeletal muscle myogenesis through ERK1/2 upon arecoline treatment. Since defects of skeletal muscle associates with several diseases, NAC can be a potent drug candidate in diseases related to defects in skeletal muscle myogenesis.

## Introduction

Skeletal musculature makes up to 40% of the human body weight [1]. Defect of skeletal musculature is associated with diseases, such as type 2 diabetes, muscular dystrophy, sarcopenia and cachexia arising from cancer, heart diseases and liver diseases [2, 3]. It is, therefore, important for health to maintain a normal function of the skeletal musculature. Skeletal muscles are formed through a process called myogenesis. Myogenesis starts from the specification of progenitor satellite cells differentiating into mononucleated myoblasts. The myoblasts then fuse into multinuclear myotubes [4]. During muscle development, myogenin activates the myogenic differentiation program [5]. Myosin heavy chain, MYH, is an actin-based motor protein and associates with muscle contraction. It plays a predominant role in specifying skeletal muscle properties [6]. Thus, myogenin and MYH are used as markers of myogenesis. Myoblast differentiation takes place not only during development but also during the regeneration of skeletal muscles after injury. Abnormal differentiation of muscles is associated with aging, type II diabetes, muscle degenerative and muscle injured diseases [7, 8]. Therefore, it will be helpful to develop drugs for the maintenance of muscle cell function and normal myogenesis for patients suffering from above diseases.

Areca nut is chewed by more than 600 million people worldwide. The principal active compound, arecoline, was shown to be genotoxic [9]. Arecoline, therefore, was classified in Group 2B as a possible carcinogen of humans on the basis of strong mechanistic evidence by the International Agency for Research on Cancer (IARC) [10]. Areca nut chewing during pregnancy was found to be associated with lower birth weight [11]. Our previous study showed that arecoline inhibited the implantation of mouse zygotes [12]. We demonstrated that arecoline inhibited myogenic differentiation of C2C12 myoblasts [13]. We also reported that arecoline inhibited acetylcholine receptor cluster formation in C2C12 myotubes [14]. These results indicate that arecoline inhibited myogenesis of C2C12 myoblasts. Therefore, C2C12 myoblasts are shown as a suitable cell model for the study of myogenetic blockage by arecoline or its recovery by drug candidates.

Arecoline can suppress cell proliferation and induces cell cycle arrest [15]. Our previous study demonstrated that arecoline inhibits the myogenic differentiation of C2C12 cells and suppresses the activation (phosphorylation) of STAT3, a transcription factor regulating myogenesis [13]. Arecoline is known to induce reactive oxygen species (ROS) and apoptosis [16]. Oxidative stress plays a critical role in the physiopathology of skeletal musculature [17]. To develop drugs that maintain the function of muscle cells and normal myogenesis, we searched for compounds capable of scavenging excess ROS.

N-acetyl-cysteine (NAC), a known scavenger of ROS, has long been used therapeutically for the treatment of an acetaminophen (paracetamol) overdose by acting as a pro-drug of hepatic glutathione (GSH) to deplete drugs through conjugation [18]. It can attenuate oxidative stress and glutathione-dependent redox imbalance in cells, such as pancreatic Rin-5F cells, and increase the viability of cocaine treated rat C6 astroglia-like cells [19, 20]. It can also increase the fertilization rate of vitrified–warmed oocytes by cleaving disulfide bonds and promoting the expansion of the zona pellucida [21]. Skeletal muscle fibers generate ROS during muscle contraction. NAC was shown to inhibit fatigue during electrical muscle activation [22]. It can also improve the function of skeletal muscles after injury [23]. NAC rescued the myotube formation in muscle-specific estrogen-related receptors knockout M-ERRγ (-/-) mice [24]. NAC scavenged excessively the arecoline-induced reactive oxygen species production and increased cell viability of normal as well as prostate cancer cells [25]. It also inhibited the arecoline-induced hypoxia inducible factor (HIF)-1α gene expression in areca quid chewing-associated oral squamous cell carcinoma (OSCC) [26]. *In vivo* studies showed that NAC can ameliorate the developmental retardation of zebrafish embryos caused by arecoline [27]. Above results indicate that NAC prevents different cells from the damage caused by arecoline. However, the effect of NAC on myoblast differentiation inhibited by arecoline was not studied as yet. We examined here the effect of NAC on the inhibited myoblast differentiation caused by arecoline and the related mechanism for this.

## Materials and methods

### Cell culture and viability analysis

Mouse myoblastic C2C12 (Bioresource Collection and Research Center, BRCR, Hsinchu, Taiwan) cells were maintained in Dulbecco’s modified Eagle’s medium (DMEM, Gibco, USA), supplemented with 10% fetal bovine serum (FBS, Gibco, USA), and antibiotics. The procedures of the cell survival assay were similar as previously described [13]. Briefly, 5×10^3^ cells (passage number <10) were cultured in 96-well plates for 24 hours and then treated with different concentrations (0, 0.1, 0.5, 1, 2, 5, 10, and 20 mM) of NAC (Actein Granules, Synmosa Biopharma Corporation, Hsinchu, Taiwan) for additional 24 or 48 hours. Twenty microliters of CellTiter 96 Aqueous One Solution Reagent (Promega, Madison, WI, USA) was added to each well and incubated at 37°C for 1 hour. Finally, the absorbance was measured at 490 nm.

### Myogenic differentiation

The procedures of myoblastic cell cultivation were following Chang et al. (2012). Briefly, myoblastic C2C12 cells (5×10^5^) were seeded in each of a 6-well plate for 24 hours. The media were changed to DMEM with 2% horse serum (HS, Gibco, USA) and at the same time treated with arecoline (Tokyo Chemical Industries, Tokyo, Japan) and NAC for 7 days. The cells were then fixed with 4% paraformaldehyde (J.T. Baker, Radnor Township, Pennsylvania, USA) for 30 min and stained with hematoxylin and eosin (H&E staining, Muto Pure Chemicals, Tokyo, Japan). The myoblast differentiation was morphologically determined by measuring the total number of multinucleated myotubes and the average number of nuclei in each myotube. The cells were imaged by a phase contrast microscope Nikon Diaphot TMD (Nikon, Nikon Corporation, Tokyo, Japan) at 100x magnification.

### Immunofluorescence

The procedures of immunocytochemistry were similar as previously described (Chang et al., 2012). Briefly, C2C12 cells were induced to form myotubes as described above. Cells were permealized in 0.1% Triton X-100 (Merck, Darmstadt, Germany)/ PBS for 5 min, blocked in 1% bovine serum albumin (BSA, Sigma)/ PBS for 1 hour, and then treated with mouse anti-MYH1/2/4/6 (Santa Cruz Biotechnology, Santa Cruz, CA) antibody for 1 hour. After washing with 0.5% Tween 20 (Sigma)/ PBS and blocking with 1% BSA/PBS, cells were treated with DyLight 594 conjugated anti-mouse secondary antibody (Abcam, Cambridge, MA, USA) for 1 hour. Cells were then stained with DAPI (4’,6-diamidino-2-phenylindole) for 15 min and visualized by fluorescence microscope ZEISS Axiovert 40 CFL (ZEISS, Carl Zeiss AG, Oberkochen, Germany) at 100x magnification. Images were processed using ImageJ and the intensity of MYH was quantified.

### Western blotting

The procedures of Western blotting were similar to those described previously [28]. Briefly, cells were harvested and lysed in RIPA buffer (50 mM Tris-HCl (Calbiochem, Merck), pH 7.6, 1 mM EDTA (Calbiochem), 150 mM NaCl (J.T. Baker), 1% Nonidet P40 (NP40, Sigma), 1% Sodium deoxycholate (Sigma), 0.1% Sodium dodecyl sulfate (SDS, J.T. Baker), and protease inhibitor cocktails (20 mM NaF (Sigma), 1mM Na_3_VO_4_ (Sigma) at 4°C for 20 minutes. The protein concentrations were determined based on the method of Bradford using Bio-Rad Protein Assay Dye Reagent Concentrate (Bio-Rad, California, USA). The 40 ug protein lysates were subjected to sodium dodecyl sulfate-polyacrylamide gel electrophoresis (SDS-PAGE) and immunoblotting. The blots were probed with Goat anti-Actin (C4) IgG_1_ (Santa Cruz, sc-47778, 1:5000 dilution), Mouse anti-MYH 1/2/4/6 IgG_1_ (F59)(Santa Cruz, sc-32732, 1:1000 dilution), Mouse anti-myogenin IgG_1_ (F5D) (Santa Cruz, sc-12732, 1:1000 dilution), Rabbit anti-ERK 1(C-16) IgG (Santa Cruz, sc-93, 1:1000 dilution), Rabbit anti-p-ERK (E-4) IgG_2a_ (Santa Cruz, sc-7383, 1:1000 dilution), followed by secondary antibodies, Donkey anti-Goat IgG-HRP (Santa Cruz, sc-2020, 1:5000 dilution), anti-Mouse IgG-HRP (Cell Signaling Technology, Inc., Danvers, MA, USA, #7076, 1:4000 dilution), anti-Rabbit IgG-HRP (Cell Signaling, #7074, 1:4000 dilution). In the blots of p-ERK appeared as two bands of 42 and 44 kDa representing p-ERK 2 and p-ERK 1, respectively. These blots were then stripped with stripping buffer (62.6M Tris pH 6.8, 2% SDS, 100 mM Beta-Mercaptoethanol) at 55°C for 15 min and reprobed with anti-Actin to show a single band at 43 kDa. The same blots were stripped again and reprobed with anti-ERK 1 to get two bands of 42 and 44 kDa. The blots were detected by ECL- chemiluminescence kit (GE Healthcare, Little Chalfont, UK). The images were exposed to x-ray films and quantified with software ImageJ.

### Statistical analysis

All results are shown as means ± SD from three independent experiments. Comparisons between groups were performed using one-way analysis of variance (ANOVA) and post-hoc Tukey HSD test. A p-value <0.05 was considered as statistically significant. The asterisks represent results of *p < 0.05 and **p < 0.01 using Tukey HSD test. The ANOVA test was done applying Statistics Kingdom.

## Results

### NAC less than 2 mM is not cytotoxic to myoblastic C2C12 cells

**Fig 1:**
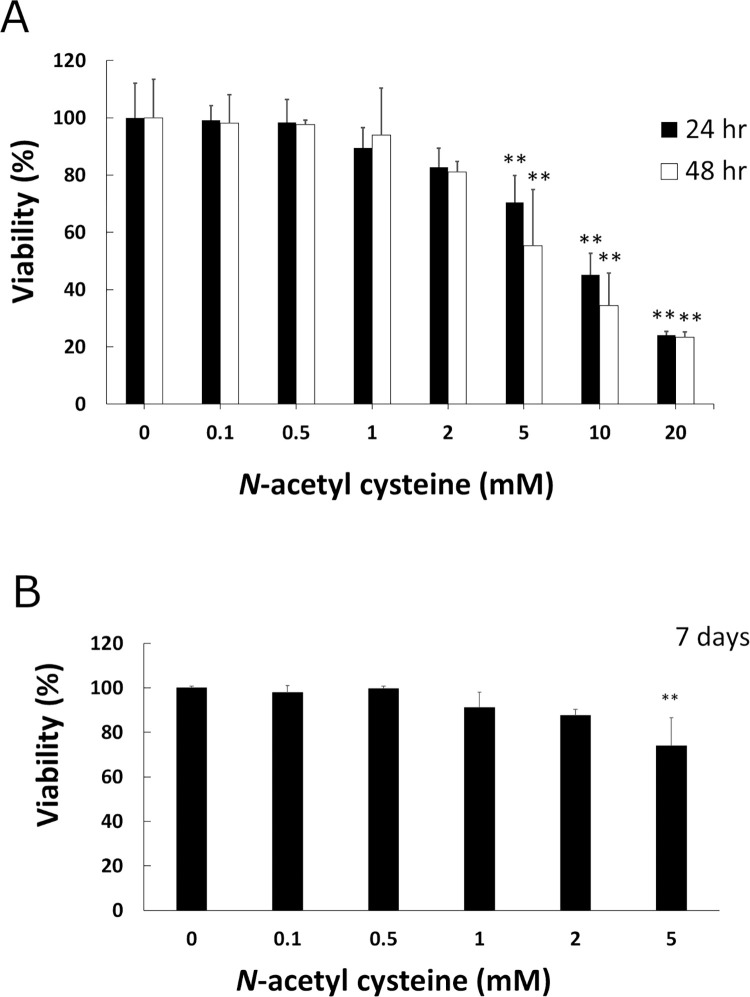
Effect of NAC on cell proliferation in C2C12 myoblasts. Cells were exposed to 0–20 mM arecoline for 24 h (solid bars) or 48 h (open bars) (A) and 7 days (B). The viability of cells was measured with CellTiter 96 Aqueous One Solution Reagent (Promega) in duplicate. The means of duplicate from three independent experiments were used for further analysis. The values of ANOVA at 24 h, 48 h and 7 days were (F_7,16_ = 56.44, P = 4.28E-10), (F_7,16_ = 26.97, P = 1.03E-07) and (F_5,12_ = 7.93, P = 1.66E-3), respectively (mean ± SD; **p < 0.01, as compared to a no-treatment control, Tukey HSD test).

Since arecoline is known to induce ROS, and NAC is a scavenger of ROS, we hypothesized that NAC plays a role in preventing arecoline-inhibited myoblast differentiation of C2C12. We first tested suitable concentrations of NAC for our studies. The cytotoxicity of NAC on C2C12 was determined by viability analysis. The results showed that NAC less than 2 mM is not cytotoxic to myoblastic C2C12 cells for 24 and 48 h (Fig 1A). Since C2C12 takes 7 days for differentiation, we further tested the viability of C2C12 with 0–5 mM of NAC for 7 days. Our results showed that NAC less than 2 mM is also not cytotoxic to C2C12 cells for 7 days (Fig 1B). We used concentrations between 0–2 mM of NAC for our future studies. We also used 5 mM NAC in some experiments as a control.

### NAC prevented the decreased number of myotubes caused by arecoline

**Fig 2:**
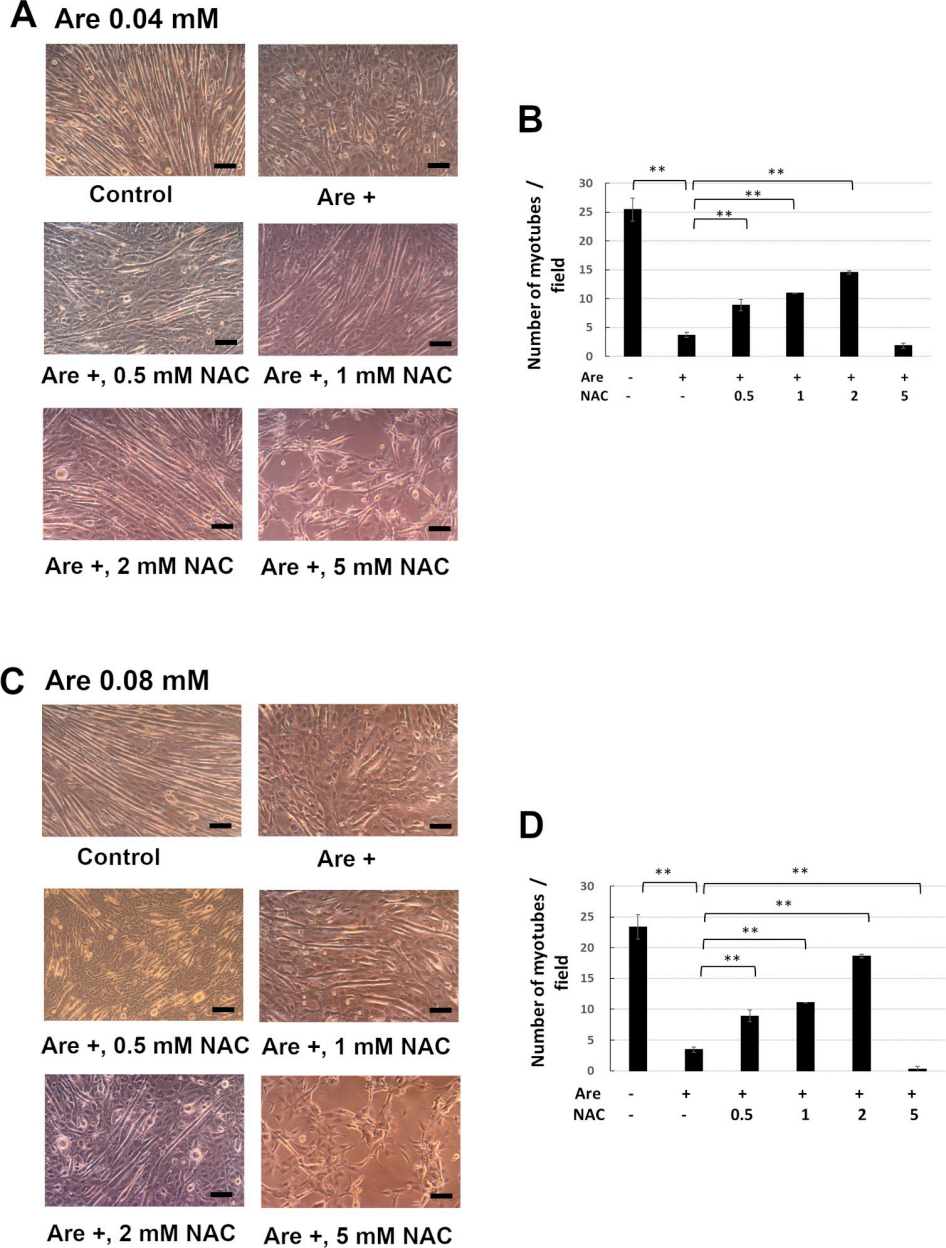
The effects of NAC on the decreased number of myotube formations caused by arecoline. C2C12 cells were cultured in differentiation medium with 0–5 mM NAC and 0.04 mM (A, B) or 0.08 mM (C, D) arecoline for 7 days. The myotubes were photographed by phase contrast microscopy (A, C). Scale bar, 100 μm. The numbers of myotubes from 30 random fields were counted (B, D). The values of ANOVA with 0.04 and 0.08 mM were (F_5,12_ = 241.90, P = 1.30E-11) and (F_5,12_ = 379.27, P = 8.99E-13) (mean ± SD; **p < 0.01, as compared to cells that were treated only with arecoline, Tukey HSD test).

In order to test the effect of NAC on the arecoline-inhibited myoblast differentiation, we measured the number of myotubes induced from C2C12 myoblastic cells. Our previous research demonstrated that 0.04 mM and 0.08 mM arecoline inhibited the myogenic differentiation of C2C12 myoblasts [13, 14]. We used these concentrations of arecoline for our experiments. The C2C12 cells were induced to differentiate for 7 days in the presence of 0–5 mM NAC and 0.04 mM arecoline (Fig 2A and 2B) or 0.08 mM arecoline (Fig 2C and 2D). The morphology of myotubes is shown in Fig 2A and 2C. The number of myotubes was significantly decreased when cells treated with arecoline were compared with those of cells without arecoline treatment (Fig 2B and 2D). However, when cells were treated with arecoline and 0.5–2 mM NAC, the number of myotubes were significantly increased compared to those cells which were treated with arecoline alone. Since 5 mM NAC is toxic to C2C12 cells, myotubes failed to form as expected. These results indicate that NAC can restore the inhibited formation of myotubes caused by arecoline at lower concentrations than 2 mM.

### NAC attenuated the decreased number of nuclei in each myotube caused by arecoline

**Fig 3:**
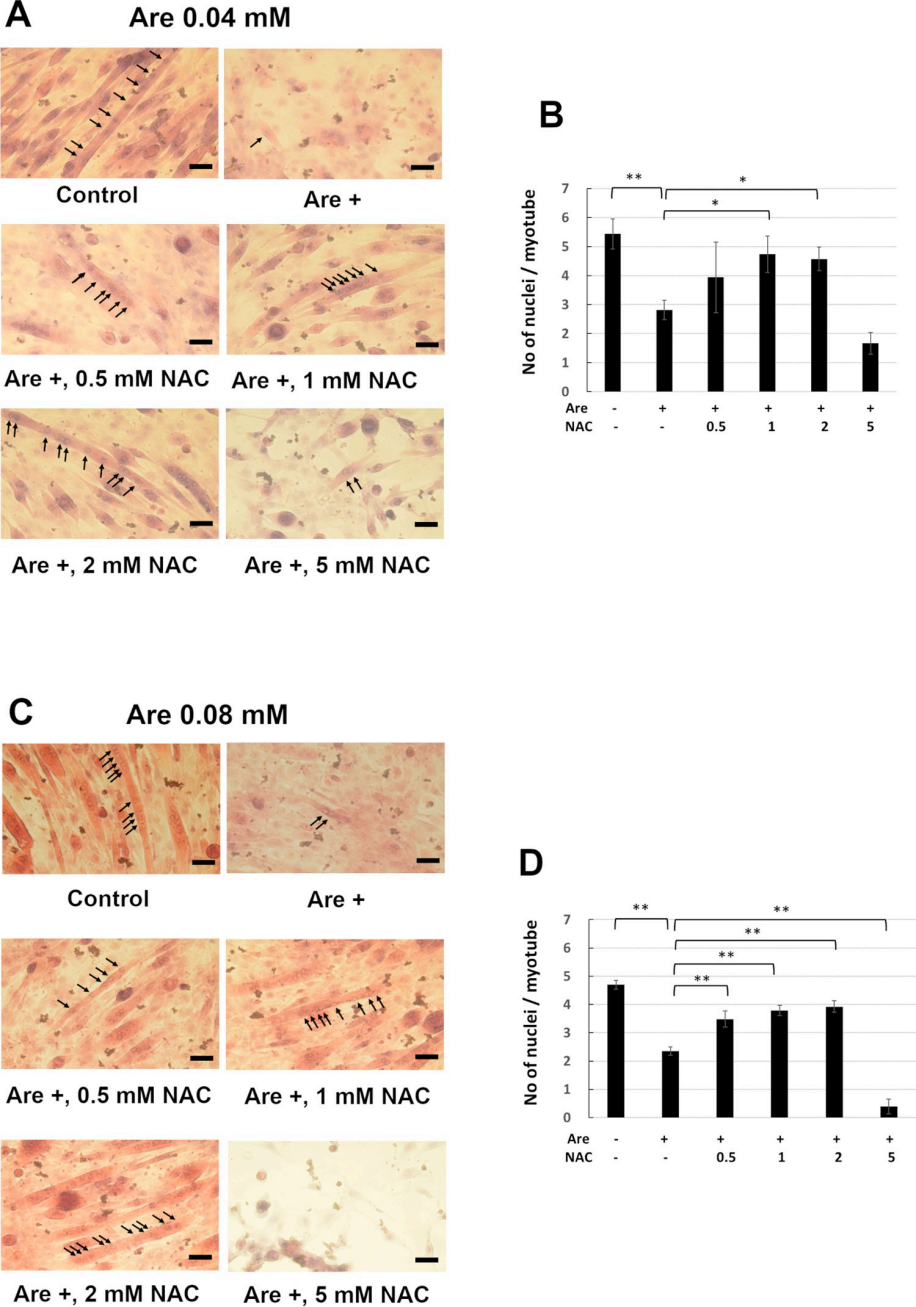
Effect of NAC on the arecoline-inhibited multinucleated myotube formation. C2C12 cells were cultured in differentiation medium with 0–5 mM NAC and 0.04 mM (A, B) or 0.08 mM (C, D) arecoline for 7 days and H&E staining. The myotubes were photographed by light microscopy (A, C). Arrows indicate the nuclei in the multinucleated myotubes. Scale bar, 100 μm. The numbers of nuclei per myotube from 30 random fields were shown (B, D). The values of ANOVA with 0.04 and 0.08 mM were (F_5,12_ = 14.51, P = 9.87E-05) and (F_5,12_ = 154.72, P = 1.83E-10). (mean ± SD; *p < 0.05; **p < 0.01, as compared to cells treated with arecoline only, Tukey HSD test).

Since NAC can prevent the arecoline-inhibited formation of myotubes, we further examined the effect of NAC on nuclei number in each myotube. The C2C12 cells were induced to differentiate for 7 days in the presence of 0–5 mM NAC and 0.04 mM arecoline (Fig 3A and 3B) or 0.08 mM arecoline (Fig 3C and 3D). The nuclei of myotubes were indicated by arrows in Fig 3A and 3C. The number of nuclei in each myotube were significantly decreased in cells treated with arecoline compared to cells without arecoline treatment (Fig 3B and 3D). However, when cells were treated with NAC, most of the nuclei numbers in each myotube were significantly increased compared to those of cells treated only with arecoline. The cells treated with 5 mM NAC failed to form myotubes with fused myoblasts. These results indicate that NAC can prevent the inhibited fusion of myoblasts in myotubes caused by arecoline.

### NAC protected the decreased expression level of myogenin from the effect of arecoline

**Fig 4:**
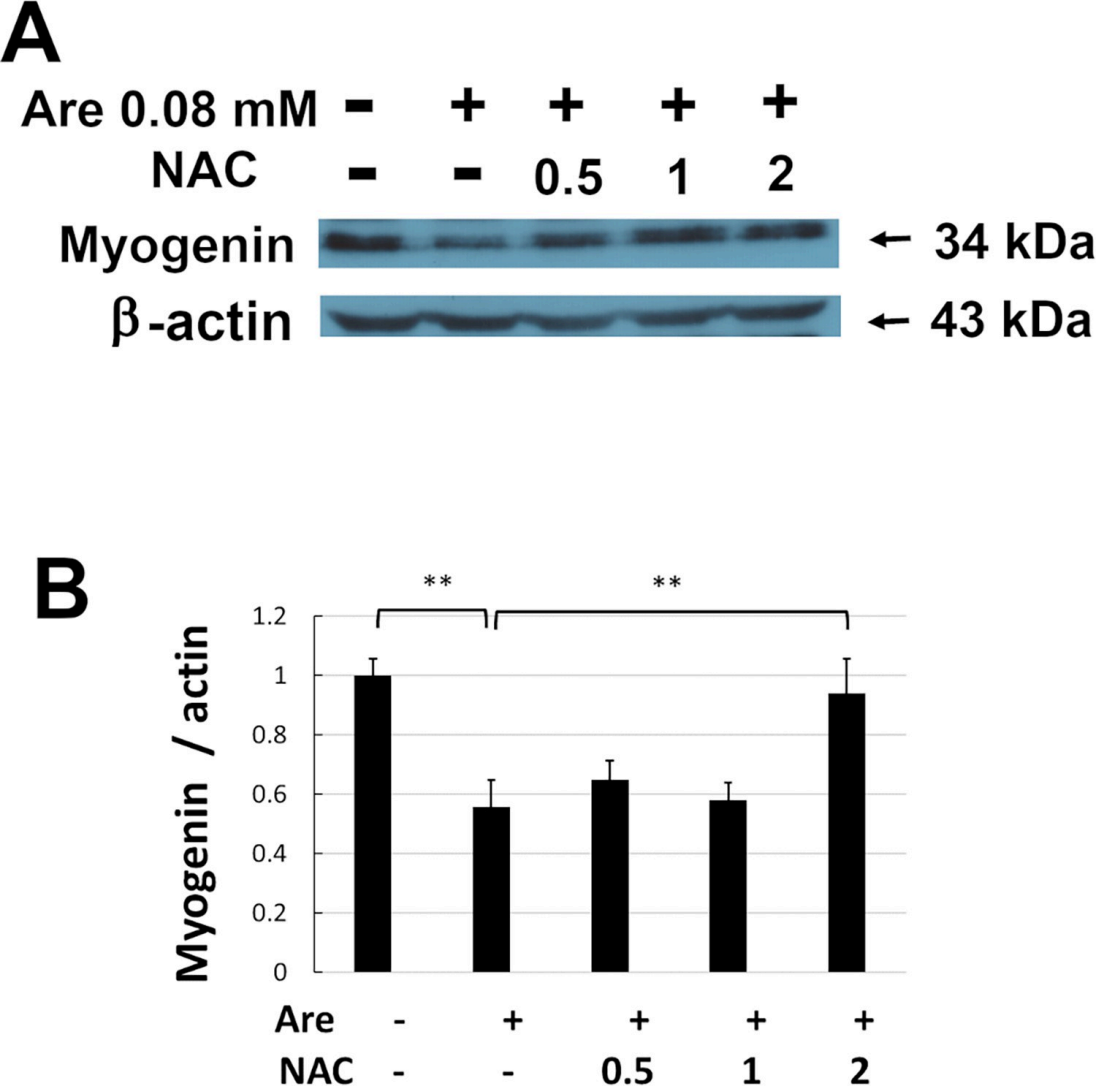
Effect of NAC on decreased myogenin expression caused by arecoline in C2C12 cells. The C2C12 cells were cultured in differentiation medium with 0–2 mM NAC and 0.08 mM arecoline for 7 days. (A) The expression of myogenin protein was detected by Western blotting. β-actin served as a loading control. (B) The band intensities of myogenin were quantified and normalized to the no-treatment control. Data from three independent experiments were used. The values of ANOVA were (F_4,10_ = 22.02, P = 6.05E-05)(mean ± SD; **p < 0.01 as compared to cells treated with arecoline only, Tukey HSD test).

Because myogenin participates in activating myogenic differentiation, the expression level of myogenin was tested by Western blotting. C2C12 cells were induced to differentiate in the presence of 0–2 mM NAC and 0.08 mM arecoline (Fig 4). The total proteins were then harvested on day 7 of differentiation and Western blotting was performed (Fig 4A). The level of myogenin was significantly increased (*p* < 0.05) by 2 mM NAC compared to cells treated only with 0.08 mM arecoline (Fig 4B). These results indicate that NAC can restore the inhibited expression of myogenin caused by arecoline.

### NAC recovered the decreased expression of MYH caused by arecoline

**Fig 5:**
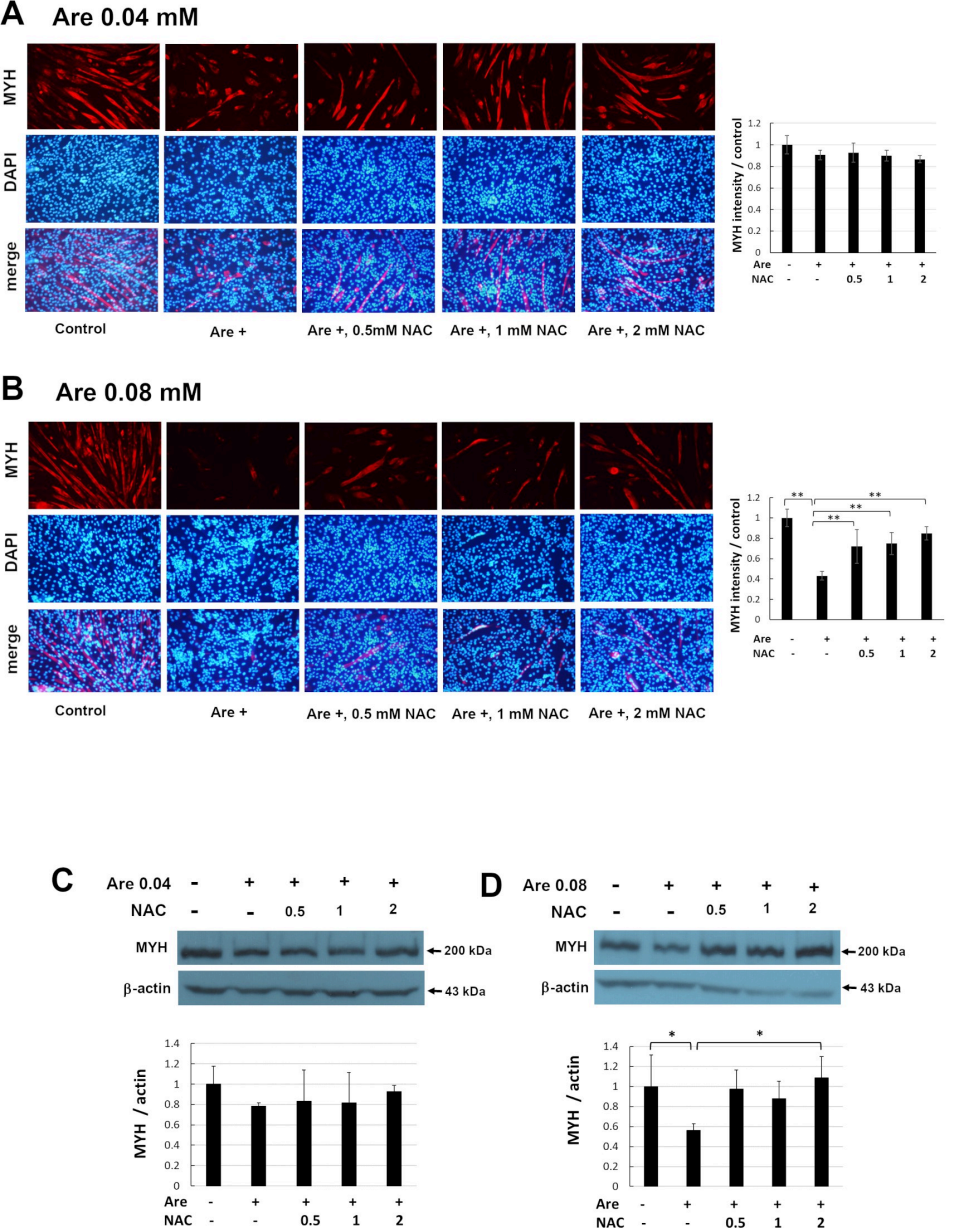
Effect of NAC on decreased MYH expression caused by arecoline in C2C12 cells. The C2C12 cells were cultured in differentiation medium with 0–2 mM NAC and 0.04 mM (A, C) or 0.08 mM (B, D) arecoline for 7 days. The distribution of MYH was examined by immunofluorescence under a fluorescence microscope (A and B, left, upper panel). Scale bar represents 100 μm at 100x magnification. Nuclei in the same field were counterstained with DAPI (A and B, left, middle panel). The images of MYH and DAPI were merged (A and B, left, lower panel). The intensities of MYH were quantified and normalized to the no-treatment control from random images of three independent experiments (Fig 5A and 5B, right panels). The values of ANOVA with 0.04 and 0.08 mM were (F_4,10_ = 2.73, P = 0.09) and (F_4,10_ = 14.49, P = 3.63E-04). The expression of MYH protein was detected by Western blotting (C and D, upper panel). β-actin served as a loading control. The band intensities of MYH from three independent experiments were quantified and normalized to no-treatment control (C and D, lower panel). The values of ANOVA with 0.04 and 0.08 mM were (F_4,10_ = 0.63, P = 0.65) and (F_4,10_ = 5.15, P = 0.02) (mean ± SD; *p < 0.05; **p < 0.01, as compared to cells treated only with arecoline, Tukey HSD test).

MYH is a marker of myogenesis specifically expressed during myogenic differentiation. We examined the expression of MYH. The C2C12 cells were induced to differentiate for 7 days in the presence of 0–2 mM NAC and 0.04 mM arecoline (Fig 5A and 5C) or 0.08 mM arecoline (Fig 5B and 5D). The distribution of MYH was presented in red color under immunofluorescence microscopy (Fig 5A and 5B, left, upper panel). The nuclei in the same pictures were stained by DAPI and turned to blue color (Fig 5A and 5B, left, middle panel). The images of MYH and DAPI were merged (A and B, left, lower panel). The intensities of MYH were quantified (Fig 5A and 5B, right panels). The results showed that the fluorescent intensity of MYH was decreased when cells were treated with arecoline as compared to cells without arecoline treatment. However, when cells were treated with arecoline and 0.5–2 mM NAC, the fluorescent intensity of MYH was increased compared to that of cells treated only with arecoline. The levels of MYH were examined by Western blotting (Fig 5C and 5D, upper panel) and the intensities of MYH were quantified (Fig 5C and 5D, lower panel). The results of Western blotting provided the same trend as that of immunofluorescence. The level of MYH was significantly decreased when cells treated with arecoline were compared with cells without arecoline treatment. However, when cells treated with arecoline and NAC, the level of MYH showed an increasing trend compared to cells which were only treated with arecoline. The level of MYH was significantly increased (*p* < 0.05) by 0.08 mM arecoline and 2 mM NAC compared to cells treated only with arecoline. These results indicate that NAC can prevent the inhibited expression of MYH caused by arecoline.

### NAC restored the decreased expression level of p-ERK caused by arecoline

**Fig 6:**
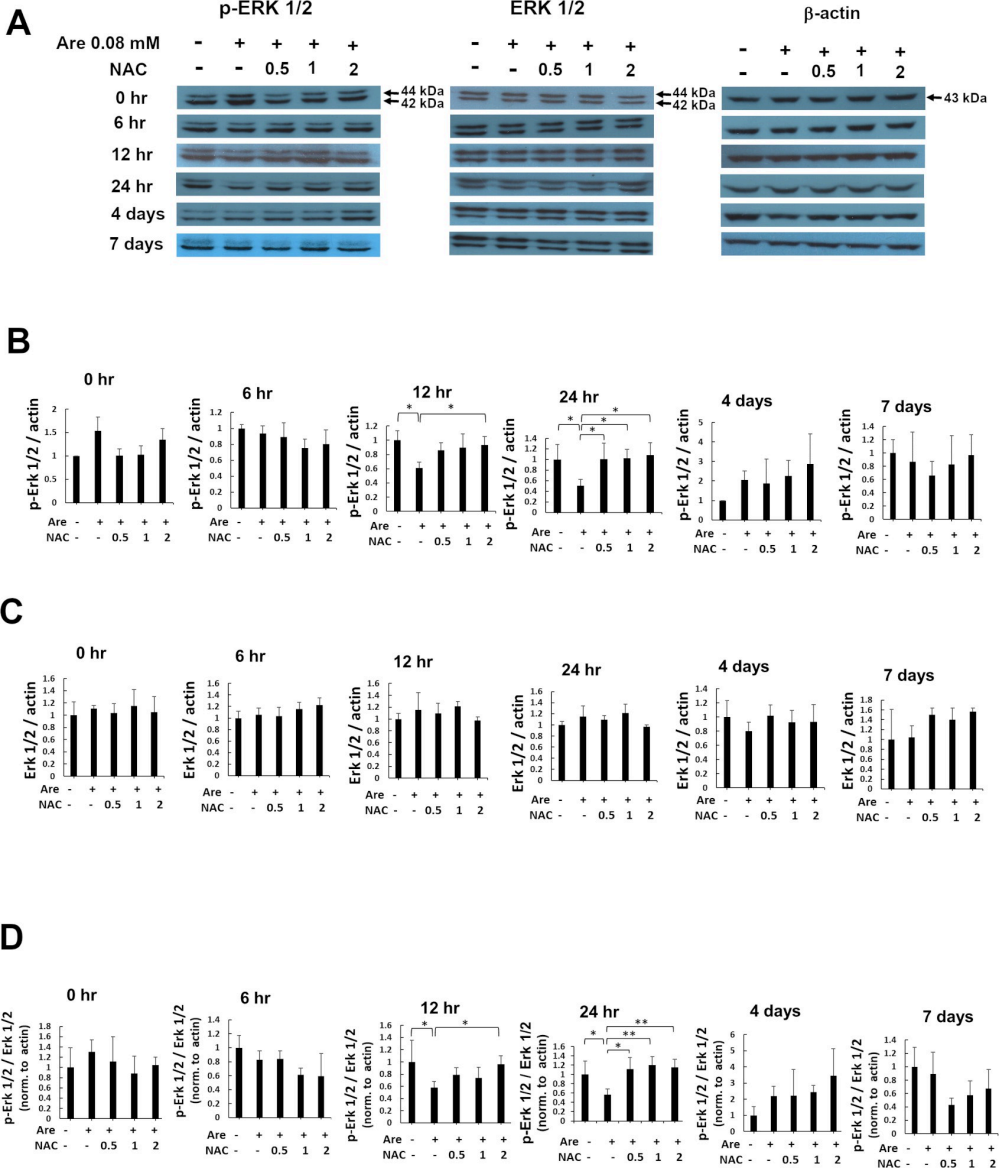
The effect of NAC on decreased ERK1/2 Tyr-204 phosphorylation caused by arecoline during myogenic differentiation in C2C12 cells. (A) The expression of Tyr-204 phosphorylated ERK1/2, ERK1/2 and β-actin proteins were detected by Western blotting at 0, 6, 12, 24 h, 4 and 7 days of myogenic differentiation in C2C12 cells treated with 0.08 mM arecoline and 0–2 mM NAC. p44:ERK1, p42:ERK2. β-actin served as the loading control. (B) The relative expression of p-ERK1/2 protein to β-actin were quantified and normalized to the no-treatment control. The values of ANOVA at 12 and 24h were (F_4,10_ = 5.03, P = 0.02) and (F_4,10_ = 5.07, P = 0.02). (C) The relative expression of ERK1/2 protein to β-actin were quantified and normalized to the no-treatment control. (D) The ratio of p-ERK1/2 to ERK1/2 from figure B and C were relative to β-actin and normalized to the no-treatment control. The values of ANOVA at 12 and 24h were (F_4,10_ = 5.54, P = 0.01) and (F_4,10_ = 7.94, P = 3.78E-03). Data from three independent experiments were presented (mean ± SD; *p < 0.05; **p < 0.01, as compared to cells treated with arecoline, Tukey HSD test).

The extracellular signal-regulated kinase (ERK1/2) is a promotor of myogenesis. C2C12 cells treated with siERK2 to knockdown the level of ERK2 protein resulted in the failure of myoblasts to fuse into multinucleated myofibers [29]. ERK1/2 does have modulatory roles in the maintenance of the slow myofiber phenotype of skeletal musculature [30]. Therefore, we hypothesized that ERK1/2 participates in the arecoline-inhibited myogenesis. Since NAC can attenuate the damage caused by arecoline, we aimed to study the role of ERK1/2 in arecoline-inhibited myogenesis attenuated by NAC. To further investigate the molecular mechanism of NAC on the inhibition of myogenic differentiation caused by arecoline, we examined the effect of NAC on the expression and phosphorylation/activation of ERK1/2 during myogenic differentiation of C2C12 cells. The C2C12 cells were induced to differentiate in the presence or absence of 0.08 mM arecoline and 0–2 mM NAC. The total proteins were then harvested at different times of differentiation. The expression of ERK1/2 and Tyr-204 phosphorylated ERK1/2, p-ERK1/2, at different times was also detected by Western blotting (Fig 6A). The amounts of p-ERK1/2 were significantly increased (p < 0.05) in cells with 0.5–2 mM NAC treatment at 24 h and 2 mM NAC treatment at 12 h compared to those of cells with only arecoline treatment (Fig 6B). However, the level of ERK1/2 were not significantly different at all of the time points (Fig 6C). The ratio of p-ERK1/2 to ERK1/2 were significantly increased (p < 0.05) in cells with 0.5–2 mM NAC treatment at 24 h and with 2 mM NAC treatment at 12 h compared to those of cells with only arecoline treatment (Fig 6D). In conclusion, these results demonstrated that the phosphorylated portion but not the total ERK1/2 was rescued by NAC during myogenic differentiation of C2C12 cells after treatment with arecoline.

## Discussion

Our previous studies demonstrated that arecoline inhibits myogenic differentiation and acetylcholine receptor cluster formation of C2C12 myoblasts [13, 14]. We used C2C12 cells treated with arecoline as a cell model. In this article we studied drugs capable of preventing the inhibition of myoblast differentiation. We found that NAC attenuated the decreased number of myotubes and the number of nuclei in each myotube which were caused by arecoline. We also found that NAC prevented the decreased expression levels of the myogenic markers, myogenin and MYH, caused by arecoline. Finally, we demonstrated that NAC restored the phosphorylated level of ERK1/2 inhibited by arecoline. Since ERK is also a positive regulator of myogenesis, our results indicate that NAC prevents the arecoline-inhibited C2C12 myoblast differentiation by activation (phosphorylation) of ERK.

We found that NAC at less than 2 mM concentration is not cytotoxic to myoblastic C2C12 cells. Actually, NAC is shown to be a safe clinical antidote [31]. It has the function of detoxifying oxidizing radicals and binding redox-active metal ions to be well known for their roles against paracetamol intoxication and as a mucolytic agent [32]. NAC is also applied in the treatment of several diseases, including polycystic ovary syndrome, premature birth and recurrent pregnancy loss, liver cancer and others [33]. It can also produce behavioral recovery after traumatic brain injury in mice and rat models [34]. For the effects on skeletal muscle, NAC significantly inhibited *amyotrophy via* antioxidant effects in rats [35]. NAC improves force capacity and fatigue properties in *ex vivo* skeletal muscles from rats after aerobic exercise [36]. NAC protects the skeletal musculature against injury induced by fatiguing contractile activity [37]. We found that NAC can promote the formation of myotubes. During myogenesis, myogenic markers are increased by NAC. These results indicate that NAC is helpful in myoblast differentiation. This is the first report to demonstrate that NAC has a beneficial effect on skeletal muscle myogenesis against the damage caused by arecoline.

Our data demonstrated that p-ERK Tyr204 phosphorylation restored by NAC treatment at 24 h during myogenic differentiation of C2C12 cells was inhibited by arecoline. These results indicate that NAC prevents arecoline-inhibited myoblast differentiation through the activation of ERK. ERK1/2 got stimulated by molecules, such as fibroblast growth factor 19, ferulic acid and EPHA2; it is further required for skeletal muscle mass regulation and myogenic differentiation [38–40]. Knockdown of ERK1/2 inhibited the differentiation of C2C12 cells [41]. The MAPK/ERK pathway involved in the skeletal muscle regeneration of adult zebrafish is regulated by Fgf [42]. MEK/ERK signaling also participated in maintaining the myogenic progenitor cells by TGFβ signaling [43]. These data indicate that ERK1/2 plays an important role in different stages of skeletal muscle myogenesis. NAC was reported to increase phosphorylated ERK to facilate neuroprotective effects [44]. Our similar results showed that NAC can regulate the phosphorylation of ERK1/2. As a scavenger of ROS, NAC removes ROS produced by arecoline through p-ERK during myogenesis.

Myogenic differentiation contains early proliferation and late differentiation stages. An ERK signaling pathway regulates the early proliferation stage of myogenic differentiation [45]. HMCA (4-hydroxy-3-methoxy cinnamic acid) was reported to have a significant effect on muscle cell differentiation. A comparison of the ratio of p-ERK/ ERK at 3 and 5 days after the treatment of C2C12 cells with 5 mM HMCA showed that p-ERK/ ERK significantly increased at 3 days but not at 5 days of treatment [46]. METTL3/14, a member of the m6A core methyltransferase complex, is required for the maintenance of muscle myogenesis. The results showed that the amount of p-ERK was increased at days 1 to 3 but decreased at days 4 to 6 after METTL3/14 treatment in C2C12 cells for skeletal muscle differentiation [47]. Time course of ERK phosphorylation levels in the culture of P19 stem cells showed that the expression of p-ERK were high at days 1 and 2 but decreased later at days 3 to 9 [48]. Our results showed that NAC increased the levels of p-ERK/ERK compared to the arecoline treatment alone at 12 and 24 h indicating that the effect of NAC was restricted to the early phase of muscle differentiation.

Areca quid chewing is a major risk factor for the generation of oral squamous cell carcinoma. NAC can downregulate the arecoline-induced ROS generation in this disease [49]. Here, we provide an example of NAC to attenuate the damage caused by arecoline. Many skeletal muscle related diseases are also related to the generation of ROS [50]. Our results demonstrated that NAC can attenuate the damage of skeletal myogenesis caused by arecoline. These results indicate that NAC has the potential for medical applications in diseases related to skeletal muscle defects.

## Conclusions

The present study provides direct evidence that NAC attenuates the damage of arecoline-inhibited C2C12 myoblast differentiation by the activation/phosphorylation of ERK1/2. Defects of skeletal muscle associates with several diseases. Our work suggests that this mechanism can be applied to clinical treatments of diseases related to defects in skeletal muscle myogenesis by NAC or NAC-related compounds.

## Supporting information

S1 Raw images (PDF)
