# Supplementary material for: N-acetyl cysteine prevents arecoline-inhibited C2C12 myoblast differentiation through ERK1/2 phosphorylation
Source: PLoS One. 2022 Jul 28;17(7):e0272231. doi: 10.1371/journal.pone.0272231 (PMC9333315; doi:10.1371/journal.pone.0272231)
Supplement: S1 Raw images — (PDF) [file pone.0272231.s001.pdf]

0.08mM Are

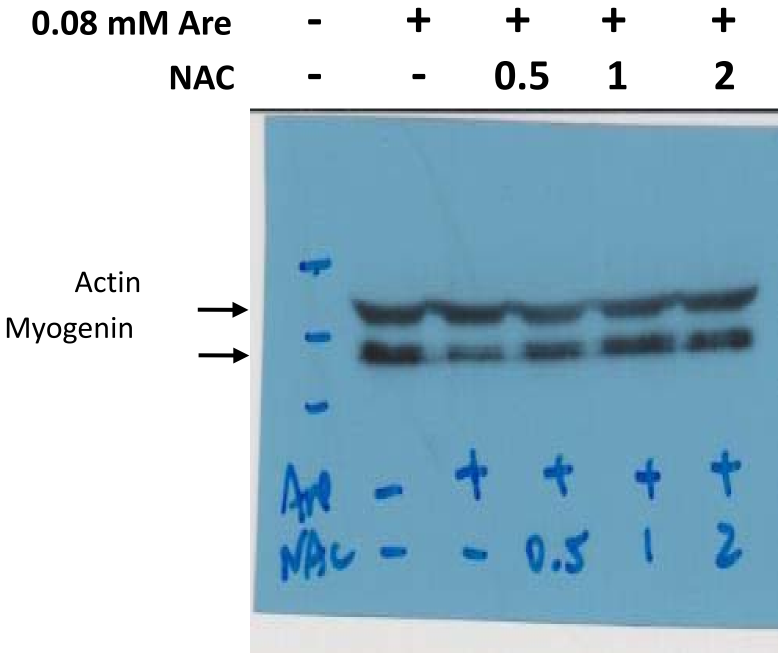

Fig.4

0.04mM Are

0.04 mM Are	-	+	+	+	+
NAC	-	-	0.5	1	2

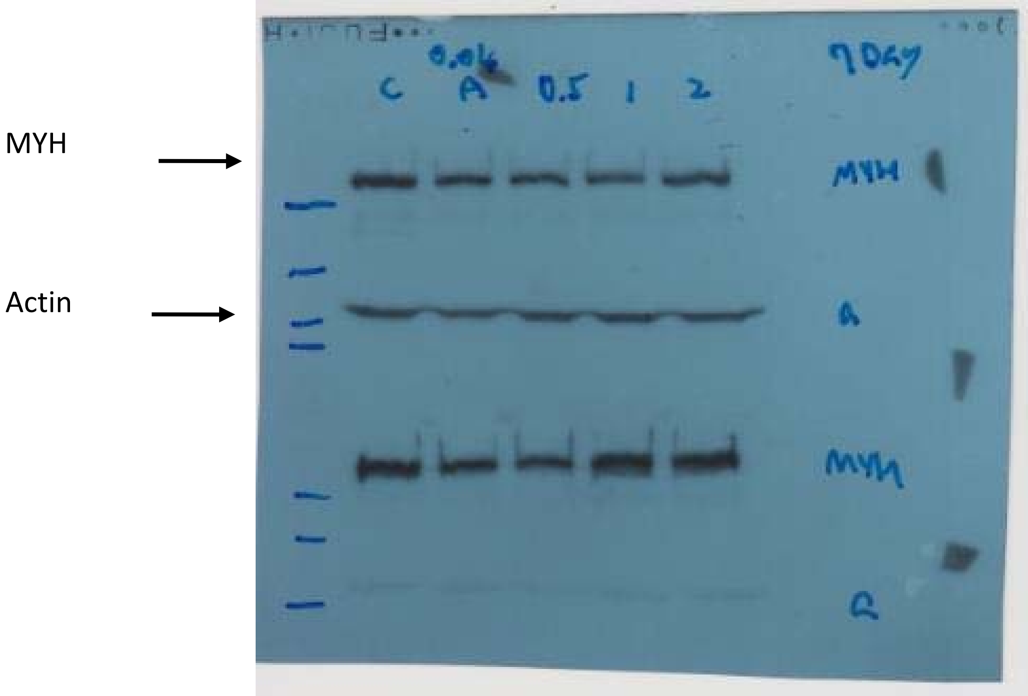

0.08mM Are

The loading order of both figs are

0.08 mM Are	-	+	+	+	+
NAC	-	-	0.5	1	2

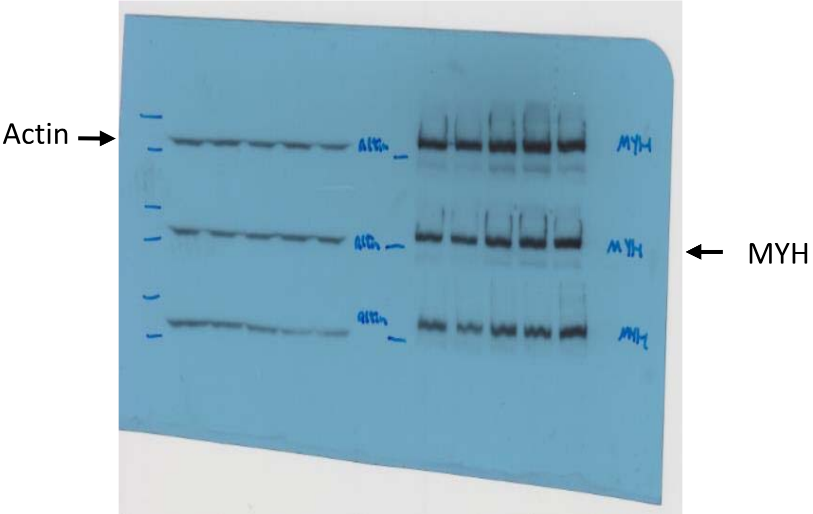

Fig.5

The loading order of all figs are

**0.08 mM Are**	**-**	**+**	**+**	**+**	**+**
**NAC**	**-**	**-**	**0.5**	**1**	**2**

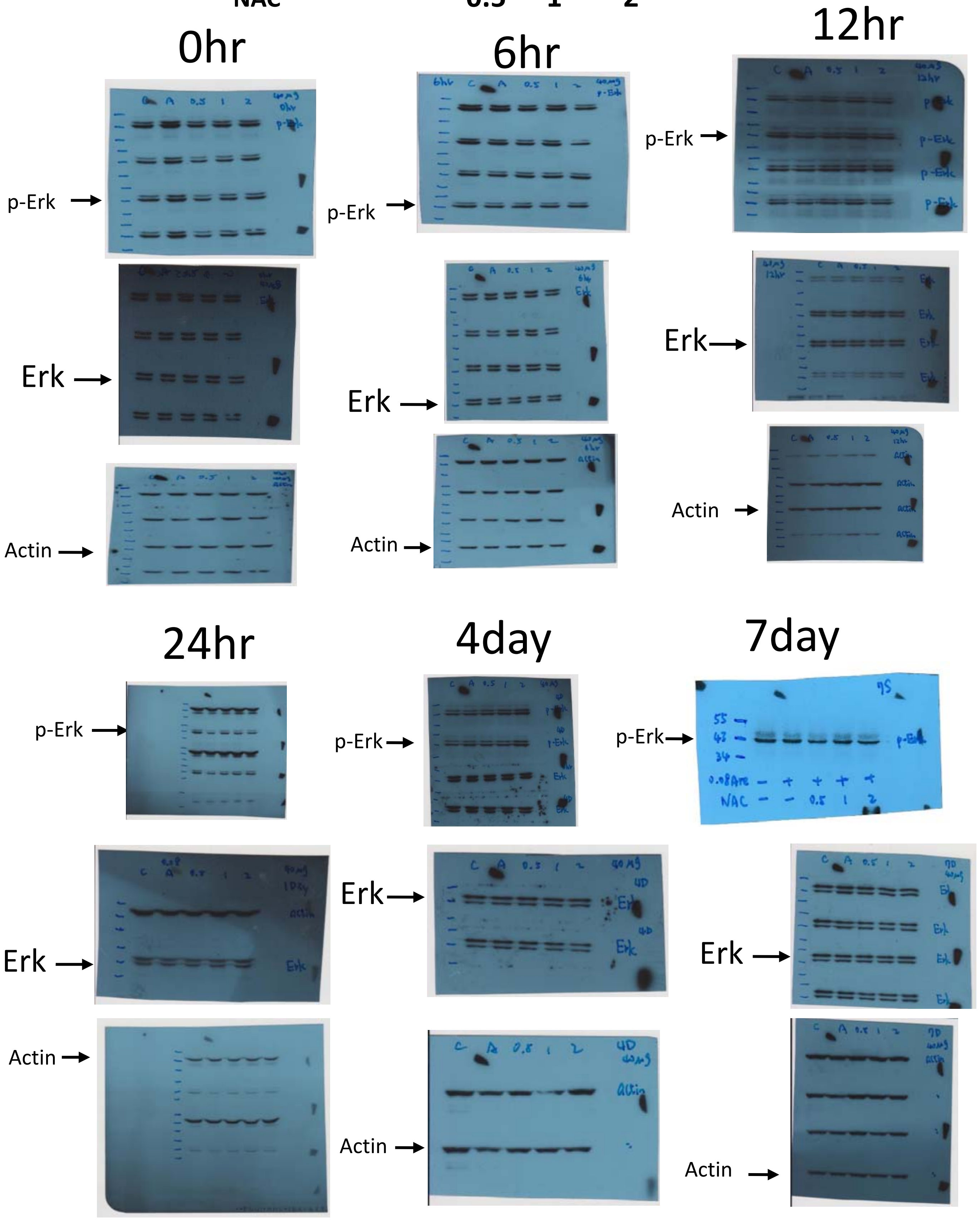

Fig.6
